# Beta-Carotene Retention and Consumer Acceptability of Selected Products Made from Two Provitamin-A Maize Varieties

**DOI:** 10.1155/2023/5575291

**Published:** 2023-12-31

**Authors:** Victor Munkhuwa, Kingsley Masamba, William Kasapila

**Affiliations:** Department of Food Science and Technology, Lilongwe University of Agriculture and Natural Resources, Lilongwe, Malawi

## Abstract

A study was carried out to determine beta-carotene retention and acceptability of selected products made from two provitamin-A maize varieties grown in Malawi, namely, MH43A and MH44A. Beta-carotene retention in the provitamin-A maize products was determined by using a “completely randomised block design” (CRBD) whereby the maize varieties (replicates) were treated as fixed blocks, and similar treatments (analytical tests) were applied in each block. Acceptability of the provitamin-A maize products was determined in 6 to 23-month-old children and their caregivers using a cross-sectional design. Results showed that in three maize products, namely, porridge, maize meal, and fermented maize beverage, there was an increase in beta-carotene, with apparent retentions of 111.13%, 170.27%, and 138.22% for MH43A and 156.50%, 207.13%, and 126.17% for MH44A varieties, respectively. Results on pregerminated maize flour produced from MH43A and MH44A maize varieties showed lower beta-carotene apparent retention values of 63.3% and 84.7%, respectively. Results on acceptability showed that most of the caregivers (47.1%) preferred porridge prepared from MH43A flour compared to porridge prepared from MH44A (30.6%) and the control variety (MH26-white maize) (22.4%). For roasted maize, roasted white maize grains (49.6%) were more preferred compared to the two provitamin-A varieties. Results on acceptability with respect to children showed that most children (63.5% and 53.7%) who tested MH43A and MH44A porridge, respectively, consumed all the porridge. Results from this study have shown that there is a high acceptability of provitamin-A maize porridges amongst children prepared from MH43A (55.5%) and MH44A (51.9%) maize varieties. The study therefore recommends that provitamin-A maize varieties should be promoted through appropriate preparation methods that ensure high beta-carotene retention to address vitamin-A deficiency.

## 1. Introduction

Traditionally, maize is a staple food eaten by the majority of people in sub-Saharan Africa including Malawi. The sub-Saharan region of Africa is a leader in the consumption of white maize, and Malawi is ranked as one of the countries with the highest consumption of white maize of 293 grams per person per day [1]. However, white maize is devoid of vitamin A and contains poor-quality protein, and its mineral composition is nutritionally inadequate [2]. Introduction of maize varieties that are genetically rich in beta-carotene would act as a cost-efficient vehicle for supplementing the masses with vitamin A. Biofortification of staple food crops increases their micronutrient density and is widely viewed as a valuable strategy for sustainably improving the nutritional status of malnourished populations [3].

The prevalence of vitamin-A deficiency (VAD) amongst preschool children in Malawi is at 3.6% [4] and is classified as a mild problem [5]. Though the percentage seems small, if left unchecked, the prevalence may progress into a severe problem. VAD has been reported to contribute to night blindness, maternal complications, and reduced body immunity against infections, all of which reduce the likelihood of survival from serious illness [5].

In Malawi, provitamin-A orange maize is associated with famine because it was once used as a safety net measure in response to the 2004 famine. A related study [1] reported that yellow maize is not popular in Africa for reasons associated with the perception of social status. The researchers observed that yellow maize is associated with food-aid programs and is perceived as being consumed only by poor people.

In 2016, the Department of Agriculture and Research Services (DARS) under the Ministry of Agriculture in Malawi [6] released two provitamin-A biofortified maize varieties, namely, MH43A and MH44A which are being propagated by Seedco and PCM agribusiness companies, respectively. Some studies [7] have previously reported the measurement of micronutrient retention as an important aspect of research on biofortified foods as high losses of micronutrients in processing and cooking reduce the nutritional value of biofortified food. Similarly, other studies have also reported that while consumers seek convenient and healthy products, they consistently rate taste as the most important factor driving consumption and, in particular, repeat purchase [8, 9].

Against this background, this study was carried out to assess beta-carotene retention levels and acceptability of selected provitamin-A maize products. The expectation is that the information obtained will be of significant use to health and agriculture research and extension workers as well as other related professions in promoting the nutrition status of the Malawi population.

## 2. Materials and Methods

### 2.1. Maize Sample Collection and Processing

**Figure 1:**
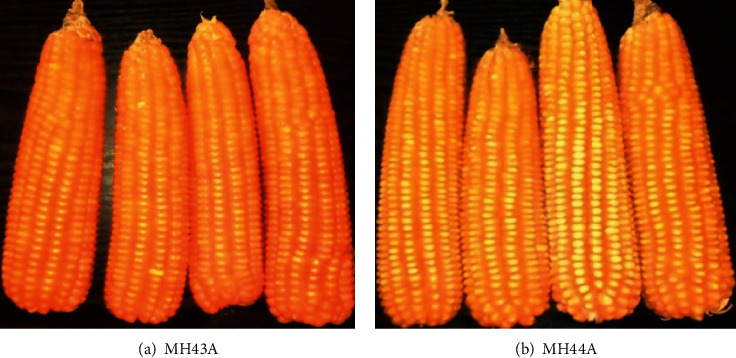
Provitamin-A maize varieties released by DARS. Source: DARS [6].

Provitamin-A maize grains of MH43A and MH44A varieties (see Figure 1) and a white maize variety of MH26 (control) were obtained from DARS at the Chitedze research station in Lilongwe. MH43A and MH44A strains are untreated flinted provitamin-A hybrid maize grains that are poundable. They are double cobbing; hence, they can be used for green/dry maize selling business as well as home consumption. MH26 maize variety is also a flint white hybrid maize variety that is devoid of beta-carotene and is a common staple variety consumed in Malawi. Chemical analysis of the maize samples was done at Lilongwe University of Agriculture and Natural Resources laboratories.

### 2.2. Determination of Beta-Carotene in Maize Products

Determination of beta-carotene content was done on whole meal maize flour, pregerminated maize flour, porridge, maize meal, and fermented maize beverage from MH43A and MH44A maize varieties using a “completely randomised block design” (CRBD). Maize varieties (replicates) were treated as fixed blocks, and similar treatments (analytical tests) were applied in each block. Laboratory analysis on the samples was done using the method of Rodriguez-Amaya and Kimura [10] as shown below.

#### 2.2.1. Sampling and Sample Preparation

MH43A and MH44A provitamin-A maize grains were processed into dry maize flour, pregerminated maize flour, porridge, maize meal, and fermented maize beverage. The samples were placed in labelled plastic sacks to reduce dehydration and were then taken to the laboratory for use within 48 hours. In the laboratory, the method of Rodriguez-Amaya and Kimura [10] was employed to prepare samples for beta-carotene extraction. The samples were packaged in aluminium foil and labelled for subsequent beta-carotene extraction.

#### 2.2.2. Extraction of Carotenoids

A portion weighing 5 g from each homogenized sample was transferred into a mortar. Then, celite weighing 3 g was added. The mixture was ground with 50 mL of cold acetone (refrigerated for 2 hours) and then filtered with suction through a Buchner funnel with filter paper. The mortar, pestle, funnel, and residue were washed with acetone, and the filtrate was recovered in the suction flask.

#### 2.2.3. Partitioning of Carotenoids

Forty milliliters (40 mL) of petroleum ether (PE) was put in a 500 mL separating funnel with a Teflon stop-cock, and acetone extract (filtrate) was added. Then, 300 mL of distilled water was added slowly along the walls of the funnel. Then, the funnel was washed three times with 200 mL of distilled water to remove residual acetone. Thereafter, the petroleum ether phase was collected in a 50 mL volumetric flask containing 15 g of anhydrous sodium sulfate to remove residual water. This procedure was done for each variety.

#### 2.2.4. Reading Using Spectrophotometer

The filtrate was put in a 1 × 1 cm glass cuvette, and absorbance readings were taken at *λ*450 nm to determine beta-carotene content using UV/Vis Spectrophotometer (CECIL CE7200) [10].

### 2.3. Beta-Carotene Apparent Retention

Apparent retention is the ratio of nutrient content in the processed food to the nutrient content in the raw food and is expressed on a dry weight basis. Apparent retention was therefore calculated, using the equation described by Murphy et al. and Pillay et al. [11, 12], which is as follows:

(1) $$\begin{array}{c} \text{Apparent retention }\left(\%\right)=\frac{\text{Nutrient content per g of processed food }\left(\text{dry basis}\right)}{\text{Nutrient content per g of raw food }\left(\text{dry basis}\right)}*100. \end{array}$$

### 2.4. Processing of Provitamin-A Maize Products

The study processed whole meal maize flour, pregerminated maize flour, porridge, maize meal, roasted maize grains, and fermented maize beverage for assessments. Sensory evaluation was done in porridge and roasted maize grains while beta-carotene analysis was done in maize products of whole meal maize flour, pregerminated maize flour, porridge, maize meal, and fermented maize beverage in triplicates to determine its apparent retention. The products were produced using locally acceptable recipes as shown below.

#### 2.4.1. Whole Meal Maize Flour

Whole meal maize flour was prepared by removing all debris from the dry maize grains followed by milling using a grinding mill (Wiley mill) with a 1 mm particle size sieve.

#### 2.4.2. Pregerminated Maize Flour

Pregerminated maize flour was obtained by firstly soaking the provitamin-A maize grains in water until it had sprouted (for approximately 96 hours at room temperature). The sprouted maize grains were then sun-dried and milled using a grinding mill into flour. The flour was now ready for laboratory analysis of beta-carotene retention and for processing of fermented maize beverage.

#### 2.4.3. Provitamin-A Maize Porridge

Porridge was obtained by firstly heating 700 mL of water in a cooking pot which was put on a source of heat. Before the water started boiling, 80 g of whole meal flour was spread on it while stirring to make it thoroughly mixed. After the mixture had started boiling evenly, the pot was covered, and the mixture was allowed to boil until it was thoroughly cooked (approximately 13 minutes). The porridge was then ready for consumption and was served warm. A sample was also taken for laboratory analysis of beta-carotene retention.

#### 2.4.4. Provitamin-A Maize Meal

Maize meal was obtained by firstly heating 700 mL of water in a cooking pot which was put on a source of heat. Before the water started boiling, 80 g of whole meal flour was spread on it while stirring to make it thoroughly mixed. After the mixture had started boiling evenly, the pot was covered, and the mixture was allowed to boil until it was thoroughly cooked (approximately 13 minutes). Thereafter, 50 g of whole meal flour was slowly added while stirring to make it thoroughly cooked. After that, the maize meal was served and was ready for consumption. A sample was also taken for laboratory analysis of beta-carotene retention.

#### 2.4.5. Fermented Maize Beverage

Fermented maize beverage was prepared by firstly heating 3.5 L of water in a pot until it was 50°C. Thereafter, 300 g of whole meal flour was gradually added to the water while stirring to make it thoroughly mixed until it had started boiling. The mixture was left to boil until it was thoroughly cooked (approximately 20 minutes). Then, the pot was removed from the heat source, and the porridge was poured in a basin and stirred for some time to cool. After it had cooled to a nonscorching point (approximately around 38°C), 400 g of pregerminated maize flour was slowly added (while stirring) followed by 2.5 L of water which was also added while stirring the mixture (for approximately 3 minutes). The mixture was then covered and left for approximately 12 hours to allow fermentation to take place. Finally, the beverage was reheated (for approximately 45 minutes) and was thereafter ready for consumption. A sample was also taken for laboratory analysis of beta-carotene retention.

#### 2.4.6. Roasted Maize Grains

Roasted maize grains were prepared by firstly selecting maize grains that were not spoilt and damaged. 500 g of maize grains that were whole and in good condition were put in the roasting pan which was later on put on the source of heat. Once the grains started roasting, the maize was kept stirred until it was thoroughly roasted and was brownish (took approximately 10 minutes). The roasted maize grains were then ready for consumption and were served warm. A sample was also taken for laboratory analysis of beta-carotene retention.

### 2.5. Sample Size and Demographic Data Collection

A total of 245 children aged 6 to 23 months old (132 from Chitedze; 113 from Mponela) and 210 caregivers (88 from Chitedze; 122 from Mponela) at Chitedze and Mponela health centers assessed the maize products for acceptability. As an exclusion criterion, children and their caregivers who were not feeling well were left out. Each age group had ≥50 subjects, which is in accordance with the accepted sample sizes for consumer acceptance and preference tests [13]. Age, sex, name of traditional authorities, and districts of the participants were recorded on designated forms.

### 2.6. Product Preference Ranking Scale with Blindfolding Technique

Blindfolding technique, which aims at blocking panellists' sight with a blindfold to exclude visual biasness in passing judgments of test samples, was used in preference ranking of maize products by caregivers. Porridge and roasted maize grains prepared from MH43A, MH44A, and white (MH26-control) maize varieties were served in different sessions for preference ranking. Under each session, caregivers were blindfolded and served with three samples of a product from each of the three maize varieties to taste and rank the samples based on their preferential varieties. The interviewer recorded the feedback on the scale, as shown in Table 1.

### 2.7. Plate Waste Scale

Plate waste scale, which measures the quantity of food left on the plate by the child, was used to assess the acceptability of provitamin-A maize porridge in 6- to 23-month-old children. Out of the 245 children that participated in the assessment, 137 children assessed MH43A while 108 children assessed MH44A maize porridge. 200 g of porridge was served to each child to be fed by its caregiver within 30 minutes, and the amount of porridge remaining on the plate was recorded. The environment was made conducive to ensure that the children were not distracted from eating. The sessions were conducted around midmorning hours to ensure that the children were neither full nor hungry of food. The collected data was filled out on the scale as shown in Table 2.

### 2.8. Likert Scale

A Likert scale, which measures people's attitude towards a sample, was used to collect complementary data to the one obtained by the plate waste scale. The scale had five points of classifications (see Table 3) which the interviewer used in assessing the child's level of satisfaction with the porridge. The child's facial expression towards the porridge was used to determine his/her satisfaction level. The interviewer recorded the feedback on the scale shown in Table 3.

### 2.9. Data Analysis

Data was entered in Microsoft Excel and exported to SPSS version 24 for analysis. Descriptive statistics were generated, and significant differences were considered at *P* < 0.05. Pearson's correlation coefficient was used to measure an association between the extent of consumption and levels of satisfaction with porridge in children.

## 3. Results and Discussion

### 3.1. Retention of Beta-Carotene in Provitamin-A Maize Products

Results of beta-carotene apparent retention in provitamin-A maize products are presented in Table 4. The results showed that in three maize products, namely, porridge, maize meal, and fermented maize beverage, there was an increase in beta-carotene, with apparent retentions of 111.13%, 170.27%, and 138.22% for MH43A and 156.50%, 207.13%, and 126.17% for MH44A varieties, respectively. When comparisons were made for pregerminated maize flour of both MH43A and MH44A, lower apparent retention values of 63.55% and 84.74%, respectively, were obtained. The differences in apparent retention percentages for similar products made from the two varieties could be attributed to the differences in genotype between the two maize varieties. A similar study also reported a significant varietal effect on beta-carotene retention after processing [14]. The authors reported that cassava varieties that had the highest beta-carotene concentration in the fresh roots did not necessarily have the highest concentration after processing.

Increases in beta-carotene concentration of the processed samples did not really reflect increases in beta-carotene content but rather reflected improved laboratory beta-carotene extraction efficiency from the processed samples. In a similar study [10], increases in carotenoid content during thermal processing were associated with greater extractability of carotenoids from processed samples. The researchers observed that carotenoids in nature are protected by cellular structures that are made up of protein. They further observed that thermal processing denatures cell wall protein and destroys cell wall structures, a scenario that facilitates the release of carotenoids from the food matrix during digestion. This culminates in greater extractability of beta-carotene in thermally processed products as compared to nonthermally processed products. This is in agreement with findings from a related study [15] that linked improvements in bioaccessibility of beta-carotene after thermal processing to the breakdown of cellulose structure of plant cells.

The reduced beta-carotene apparent retention of <100% in pregerminated maize flour indicates that soaking of maize reduces the beta-carotene concentration in its products. This is in agreement with results from similar studies [16, 17] that reported reductions in beta-carotene, phytic acid, total polyphenol, iron, and ash content (*P* < 0.05) as the soaking time increased. Furthermore, a similar study [18] also observed that soaking of samples increase water content in the cellulose which does not only behave as bulk water medium but also actively participates in the cellulose structure and function. This stabilizes and makes the cell wall rigid and tough and hence reduces the extractability of beta-carotene during chemical analysis. This is in line with the current findings as the pregerminated maize was soaked in water for 96 hours, and there was a subsequent reduction in beta-carotene of MH43A (63.55 percent) and MH44A (84.74 percent) maize varieties.

Fermentation of maize porridge to make beverages does not adversely affect beta-carotene concentration as the product has increased apparent retention of 138.22% and 126.17% for both MH43A and MH44A maize varieties, respectively. This is in agreement with the results of a similar study [19] which also reported that fermentation does not adversely affect the retention of beta-carotene in porridges prepared with high beta-carotene maize.

### 3.2. Acceptability of Provitamin-A Maize Products

#### 3.2.1. Preference Ranking of Maize Products by Blindfolded Caregivers

Results on preference ranking of maize porridge and roasted maize grains by blindfolded caregivers are presented in Table 5. Most of the caregivers (47.1%) who tested porridge from the three maize varieties ranked MH43A as their first choice. Porridge made from MH44A variety came second followed by white maize (MH26) variety. This means that if made available, caregivers would be willing to feed their children with the porridge, hence facilitating their improvements in vitamin-A status and reductions in VAD syndromes. These results are in agreement with findings from a related study [20] which reported that yellow provitamin-A maize soft porridge was as acceptable as white maize soft porridge to infant caregivers from the rural areas of the uMgungundlovu District of KwaZulu-Natal in South Africa.

On the other hand, most of the caregivers (49.6%) who tested roasted maize grains ranked white maize as their first choice with MH44A and MH43A as their second and third choice, respectively. This could be attributed to their familiarity with sensory properties of white maize as it was their usual variety of consumption. A similar study [21] stated that sensory attributes, especially texture, are a major determinant of the acceptance of provitamin-A biofortified maize by consumers.

### 3.3. Extent of Consumption and Level of Satisfaction with Provitamin-A Maize Porridge

Results on the extent of consumption and level of satisfaction with provitamin-A maize porridge in 6- to 23-month-old children are presented in Table 6. Most children (63.5%) who tested MH43A maize porridge consumed all the porridge that they were served with. Similarly, of the children who tested MH44A maize porridge, the majority (53.7%) also consumed all the porridge that they were served with. Furthermore, the majority of the children (55.5% and 51.9%) showed that they were very satisfied with MH43A and MH44A maize porridges, respectively.The high percentages of satisfaction in the children can be directly linked to the high percentages of children who consumed at least three-quarters of the porridge that they were served with. This shows that just as their caregivers, the children also liked the orange porridge, and if used as a complementary food, it could be a suitable vehicle to provide vitamin A to their age group. The favoring of orange maize porridge by the children could be attributed to improvements in orange maize porridge attributes as compared to in white maize porridge. A similar study [22] reported that biofortified maize porridge is finer in texture and has an intense cooked maize flavour and aroma than white maize porridge. Furthermore, another related study [23] also reported that provitamin-A biofortified maize varieties are not as hard as white maize varieties and are as such more suitable to making stiff porridge than the white maize since porridge requires grain with fairly low hardness. These results are in agreement with results from a study which reported that preschool children preferred yellow maize to white maize porridge [24].

### 3.4. Correlation between Extent of Consumption and Level of Satisfaction with Porridge

Results on correlation between extent of consumption and level of satisfaction with porridge in 6- to 23-month-old children are presented in Table 7. According to the results, there is a strong positive linear relationship between the amount of porridge consumed and the level of satisfaction with a correlation coefficient of *r* = 0.971 and *r* = 0.996 for MH43A and MH44A maize varieties, respectively. This confirms the assumption that consuming more porridge is highly associated with being very satisfied as portrayed by the facial expressions of the children when eating the porridge. Provitamin-A maize porridge has shown to be liked by 6- to 23-month-old children as such can form a better complementary food which could be high in beta-carotene and have an improved texture and flavour.

## 4. Conclusions and Recommendation

Results of this study have shown that thermal-processed maize products of porridge, maize meal, and fermented maize beverage had increased beta-carotene content with apparent retentions of greater than 100% for both MH43A and MH44A varieties. This has demonstrated that thermal-processed provitamin-A maize products are good sources of bioaccessible beta-carotene which is key in boosting the body's vitamin-A status and immunity. The findings have also shown that provitamin-A maize porridge is acceptable in both children aged 6 to 23 months old and their caregivers. Stakeholders should promote the growing of provitamin-A maize to ensure increased access and dietary incorporation of the orange varieties by consumers. Stakeholders should also promote complementary feeding of 6- to 23-month-old children with provitamin-A maize products to ensure improved vitamin-A status and decreased prevalence of VAD syndromes amongst their age categories.

## Figures and Tables

**Table 1:** Preference ranking scale.

No.	Name of mother/caregiver	Age	Maize varieties (please tick your preferred choice where appropriate)		
			White maize (MH26)	Orange maize (MH43A)	Orange maize (MH44A)
1					

**Table 2:** Plate waste scale.

No.	Name of child	Age in months	Sex		Amount of porridge consumed					Comments
					0	1/4	1/2	3/4	All	
1			M	F	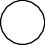	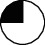	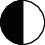	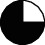	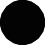	

**Table 3:** Likert scale.

Name of child:		Age in months:		
How satisfied is the child with the porridge?				
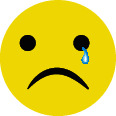	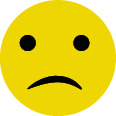	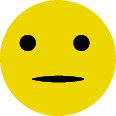	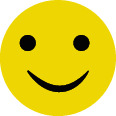	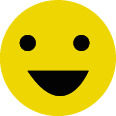
Very unsatisfied	Unsatisfied	Neutral	Satisfied	Very satisfied

**Table 4:** Beta-carotene content and apparent retention in provitamin-A maize products.

Maize products	MH43A		MH44A	
	Beta-carotene content (*μ*g/100 g)	Apparent retention (%)	Beta-carotene content (*μ*g/100 g)	Apparent retention (%)
Dry flour	19.77 ± 0.09^a^		16.70 ± 0.45^a^	
Pregerminated maize flour	12.57 ± 0.24^b^	63.55	14.15 ± 0.61^b^	84.74
Porridge	21.97 ± 0.14^c^	111.13	26.13 ± 0.38^c^	156.50
Maize meal	33.67 ± 0.29^a^	170.27	34.59 ± 0.38^b^	207.13
Fermented maize beverage	27.33 ± 0.16^b^	138.22	21.07 ± 0.07^b^	126.17

Means with different superscripts within the same row are significantly different (*P* < 0.05).

**Table 5:** Preference ranking of maize products by blindfolded caregivers.

Maize varieties	Roasted maize grains (*N* = 125)		Porridge (*N* = 85)	
	%	Rank	%	Rank
White maize (MH26-control)	49.6	1	22.4	3
Orange maize (MH43A)	23.2	3	47.1	1
Orange maize (MH44A)	27.2	2	30.6	2

**Table 6:** Extent of consumption and level of satisfaction with provitamin-A maize porridge (200 g) in 6- to 23-month-old children.

Amount of porridge consumed	MH43A (%) *N* = 137	MH44A (%) *N* = 108	Level of satisfaction	MH43A (%) *N* = 137	MH44A (%) *N* = 108
Zero	1.5	3.7	Very unsatisfied	1.5	2.8
Quarter	3.6	4.6	Unsatisfied	1.5	6.5
Half	9.5	10.2	Neutral	10.2	13.0
Three-quarters	21.9	27.8	Satisfied	31.4	25.9
All	63.5	53.7	Very satisfied	55.5	51.9

**Table 7:** Correlation between extent of consumption and level of satisfaction with porridge in children.

		MH43A		MH44A	
		Consumption	Satisfaction	Consumption	Satisfaction
Consumption	Pearson's correlation	1	.971^∗∗^	1	.996^∗∗^
	Sig. (2-tailed)		.006		.000
	*N*	5	5	5	5

Satisfaction	Pearson's correlation	.971^∗∗^	1	.996^∗∗^	1
	Sig. (2-tailed)	.006		.000	
	*N*	5	5	5	5

^∗∗^Correlation is significant at the 0.01 level (2-tailed).

## Data Availability

Data supporting the conclusions made in this manuscript has been uploaded online through http://Zenodo.org and has been cited as follows: Munkhuwa, Victor (2022); beta carotene apparent retention dataset, Likert scale dataset, and preference ranking scale dataset; Zenodo (doi:10.5281/zenodo.7180722) [25].
